# Evaluation of the Effectiveness of BNT162b2 Primary Vaccination and Booster Dose to SARS-CoV-2 in Eliciting Stable Mucosal Immunity

**DOI:** 10.3390/biomedicines10102430

**Published:** 2022-09-29

**Authors:** Alessandro Lambiase, Marta Sacchetti, Fabiana Mallone, Paola Tirassa, Antonio Greco, Antonio Angeloni, Antonella Polimeni

**Affiliations:** 1 Department of Sense Organs, Sapienza University of Rome, 00161 Rome, Italy; 2 Department of Biochemistry and Cell Biology, IBBC-CNR, 00185 Rome, Italy; 3 Department of Experimental Medicine, Sapienza University of Rome, 00161 Rome, Italy; 4 Department of Oral and Maxillofacial Sciences, Sapienza University of Rome, 00161 Rome, Italy

**Keywords:** ocular mucosal immunology, ocular mucosal homeostasis, RNA vaccines, SARS-CoV-2, antibodies

## Abstract

The waning effectiveness of the primary vaccination for SARS-CoV-2 led to administration of an additional booster dose (BD). The efficacy of the BD in stimulating humoral systemic immune response is well established, but its effectiveness on inducing mucosal immune reaction has not yet been reported. To address this issue, we evaluated SARS-CoV-2-specific antibody responses in the serum, saliva, and tears after BNT162b2 (Pfizer/BioNTech, New York, NY, USA) vaccination and BD, as well as after SARS-CoV-2 infection. After two doses of BNT162b2 vaccine, we observed specific serum IgG in 100% and IgA in 97.2% of subjects, associated with mucosal response in both salivary samples (sIgA in 97.2% and IgG(S) in 58.8%) and in tears (sIgA in 77.8% and IgG(S) in 67.7%). BD induced a recovery of the systemic humoral response and of tear sIgA when compared to 6 months of follow-up titers (*p* < 0.001; *p* = 0.012). However, sIgA levels in both tears and saliva were significantly lower following BD when compared to patients with prior SARS-CoV-2 infection (*p* = 0.001 and *p* = 0.005, respectively). Our results demonstrated that administration of BD restored high serum levels of both IgG and IgA but had a poor effect in stimulating mucosal immunity when compared to prior SARS-CoV-2 infection.

## 1. Introduction

Vaccination against severe acute respiratory syndrome coronavirus 2 (SARS-CoV-2), the causative agent of coronavirus disease 2019 (COVID-19), has shown high efficacy in preventing COVID-19 disease and improving the outcome of the infection [1]. The evidence of decline in vaccine-induced immunity over time led to the booster dose (BD) vaccination, which has been shown to be effective in inducing the recovery of systemic immunity, but its effect on the mucosal immune reaction has not yet been investigated [2,3].

Increasing evidence has shown the crucial role played by mucosal immune response against SARS-CoV-2 infection [4,5]. In fact, SARS-CoV-2 infection induces both systemic and mucosal antibody response with an increase in serum immunoglobulin (Ig)-G and mucosal soluble (s)IgA production in salivary, nasal, tear, and bronchoalveolar lavage fluids [6,7,8]. Ejemel et al. demonstrated that SARS-CoV-2-specific IgA is capable of neutralizing the virus at mucosal surfaces by binding to the virus spike protein and competitively blocking ACE2 receptor binding at the cell surface [4]. In addition, serum IgA response was found to be significantly correlated with disease severity [9,10,11]. Interestingly, Randad et al. demonstrated that the anti-SARS-CoV-2 sIgA serum titer correlates with the sIgA salivary levels, suggesting that quantification of serum anti-SARS-CoV-2 IgA may provide information on mucosal immunity [12]. 

It is well known that natural infection and systemic vaccination may induce different immune responses at systemic and mucosal levels, and that injectable vaccines generally induce poor mucosal sIgA responses [13]. To date, few studies have evaluated mucosal immune response after primary SARS-CoV-2 vaccination, showing contrasting results on whether these vaccines are able to induce sIgA response or not, while no data regarding the different IgG/IgA response following SARS-CoV-2 infection and vaccination are available [14,15,16,17,18].

To cover this question, our study evaluated and compared the SARS-CoV-2-specific antibody responses in the serum, salivary, and tear samples of subjects receiving primary vaccination cycle and BD with the mRNA BNT162b2 (Pfizer/BioNTech, New York, NY, USA) vaccine, as well as of subjects recovered from SARS-CoV-2 infection. 

## 2. Results and Discussion

**Figure 1:**
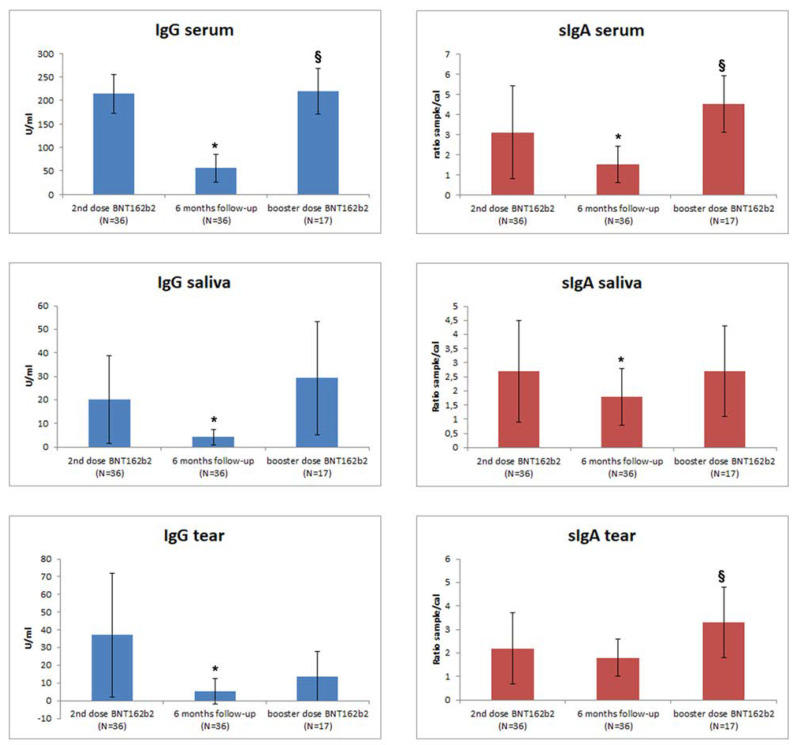
IgG and IgA mean levels ± SD in serum, saliva, and tears of subjects receiving BNT162b2 vaccination. The trend following first vaccination, 6 months of follow-up, and after BD is shown. Descriptive statistics are reported in the text. * *p* < 0.05 statistically significant versus second dose of vaccination group; § *p* < 0.05 statistically significant versus 6-month follow-up.

As expected, two doses of BNT162b2 vaccination induced humoral systemic immune response, as shown by IgG positivity in all subjects (serum titer: 214 ± 41 U/mL) and IgA positivity in 97.2% (serum titer: 3.1 ± 2.3 Ratio sample/cal) of cases (Figure 1). Notably, mucosal immunity was also stimulated by two doses of BNT162b2 vaccination. Specifically, 97.2% of individuals were positive for sIgA in salivary samples (2.7 ± 1.8 ratio sample/cal) and 77.8% in tears (2.2 ± 1.5 ratio sample/cal). IgG(S) were also detectable in 58.8% (20.2 ± 18.6 U/mL) of salivary samples and in 67.7% of tears (37.1 ± 45.1 U/mL). We observed that IgG and IgA serum concentrations were significantly correlated (*p* = 0.019; R = 0.389) and that IgA serum titer was significantly correlated with the salivary (*p* < 0.001; R = 0.734) and tear (*p* < 0.001; R = 0.644) sIgA levels.

At 6 months of follow-up, a significant waning of humoral systemic and mucosal immune response was observed in vaccinated subjects. Specifically, both IgG and IgA were decreased in serum (IgG = 55.7 ± 30.2 U/mL and IgA = 1.5 ± 0.9 ratio sample/cal; both *p* = 0.001) and saliva (IgG = 4.3 ± 3.3 U/mL and IgA = 1.8 ± 1 ratio sample/cal; *p* = 0.001 and *p* = 0.032, respectively) when compared with values after primary vaccination, while tears showed a significant decrease in IgG (5.3 ± 7.1 U/mL, *p* = 0.004) but not of IgA levels (1.8 ± 0.8 ratio sample/cal) (Table 1).

BD induced a recovery of the systemic humoral response (IgG serum titer: 66.7 ± 31.1 U/mL vs. 219.8 ± 49, *p* < 0.001, and IgA serum titer: l.7 ± 1 vs. 4.5 ± 1.4 ratio sample/cal, *p* < 0.001). A significant increase in tear sIgA levels was also observed when compared with 6 months follow-up (sIgA tear titer: 2 ± 0.9 vs. 3.5 ± 1.5 ratio sample/cal, *p* = 0.012), while sIgA salivary levels did not show a significant increase (sIgA salivary titer: 2.2 ± 1.2 vs. 2.7 ± 1.6 ratio sample/cal) (Table 2). 

**Figure 2:**
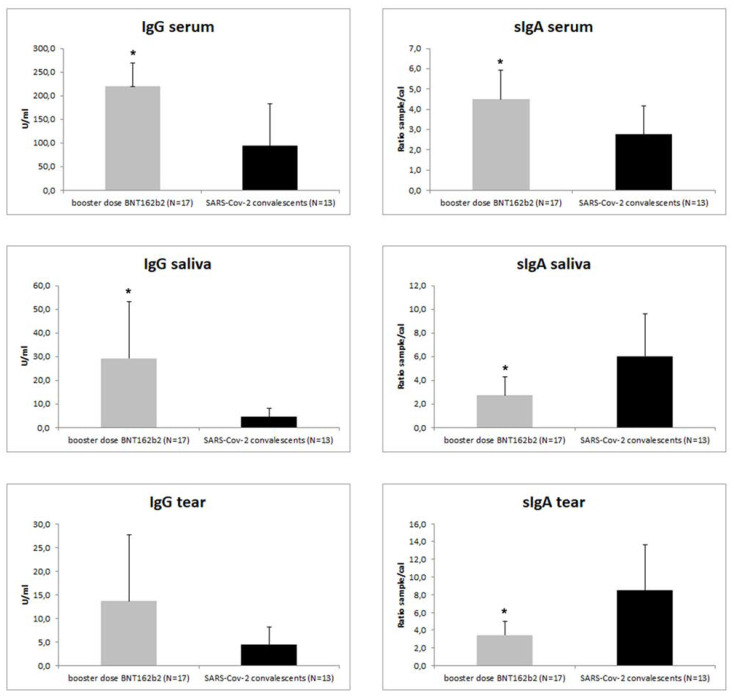
Level of IgG and IgA levels in serum, saliva, and tears of SARS-CoV-2 patients and subjects receiving booster BNT162b vaccination. Descriptive statistics is reported in the text. * *p* < 0.05 statistically significant versus the SARS-CoV-2 patient group.

The comparison between the IgG/IgA response in serum, saliva, and tears following COVID-19 infection and vaccination is shown in Figure 2. The vaccine BD induced significantly higher levels of both serum IgG (219.8 ± 49 U/mL) and IgA (4.5 ± 1.4 ratio sample/cal) levels when compared to patients with prior SARS-CoV-2 infection (IgG: 95.1 ± 88.3 U/mL, *p* = 0.016; IgA: 2.8 ± 1.4 ratio sample/cal, *p* = 0.030), but significantly lower levels of sIgA were observed in both tears (3.5 ± 1.5 ratio sample/cal, *p* = 0.001) and saliva (2.7 ± 1.6 ratio sample/cal, *p* = 0.005) (Figure 2). 

Patients with prior SARS-CoV-2 infection showed positivity for IgG and IgA in serum (95.1 ± 88.3 U/mL and 2.8 ± 1.4 ratio sample/cal, respectively). In this group of subjects, sIgA (6 ± 3.6 ratio sample/cal) but not IgG positivity was observed in salivary samples. Similarly, sIgA was present in tears (8.5 ± 5 ratio sample/cal), while IgG was positive only in two subjects (Table 3). 

RT-PCR assay for detection of SARS-CoV-2 RNA in tear samples yielded negative results in all vaccinated and previously SARS-CoV-2-infected subjects. This confirms recent data that report virus detection in tears occurring during the early stages of COVID-19 disease [19].

Our results, showing the presence of sIgA in tears and saliva of patients following anti-SARS-CoV-2 vaccination, appear in contrast with the classic dogma that systemic immunization against mucosal pathogen, such as COVID-19, induces strong production of serum antibodies but it is poorly able to stimulate mucosal immune response [13]. Moreover, the fact that systemic immunization against SARS-CoV-2 is also able to elicit a mucosal immune response might be particularly relevant since the sites of virus entry are nasopharyngeal or ocular mucosae, and sIgA, together with tissue resident memory T cells are the first line against virus entry and prompt protection upon viral antigen re-challenge [20,21,22].

Further, while it is clearly demonstrated that SARS-CoV-2 infection induces sIgA production in tears and saliva [7,8], contrasting evidence are available on the ability of systemic vaccination to stimulate the production of sIgA [14,15,16,18,23]. For example, Selva et al. [14] and Piano Mortari et al. [15] did not find sIgA changes in tear and salivary samples following BNT162b2 vaccination, while other studies reported the presence of salivary sIgA in 50% to 100% of vaccinated subjects [16,18,23,24]. Our results demonstrated that the BNT162b2 vaccine induces the presence of specific SARS-CoV-2 spike protein peptide IgA, not only in the serum but also in saliva (about 97% of subjects) and tears (about 77% of subjects), and that the BD was highly effective in restoring systemic humoral immunity and the IgA levels in tears, but not in saliva. 

In addition, we found that natural immunization with COVID-19 infection induced a significantly higher sIgA response in both tears and saliva when compared to the BD. These findings are in line with the ongoing evolution of the SARS-CoV-2 pandemic that show that an increasing number of breakthrough infections are being detected among vaccinated subjects in spite of the good efficacy of vaccine boosters to prevent severe COVID-19 disease [25,26,27]. 

## 3. Methods

We enrolled 36 BNT162b2 vaccine recipients aged 37 ± 13 years, 12 males and 24 females, as well as 13 patients with prior SARS-CoV-2 infection—mean age 29 ± 12 years, 6 male and 7 female. 

Isotype-specific antibody response anti-spike S1 IgA (Anti-SARS-CoV-2 ELISA IgA, manual ELISA; Euroimmun, Lübeck, Germany) and anti-spike RBD IgG (SARS-CoV-2 S1/RBD IgG ELISA, IBL International GmbH, Tecan Group) to SARS-CoV-2 were analyzed in the serum, salivary, and tear samples of all subjects. 

Antibody levels were evaluated at three time points in vaccinated subjects: 15 ± 2 days after the second dose of BNT162b2 (*n* = 36), 6 months after the second dose (*n* = 36), and 15 ± 2 days after receiving BD (*n* = 17, mean age 35 ± 13.2 years, 5 males, 12 females) of BNT162b2. All vaccinated subjects included in this study tested negative for both SARS-CoV-2 presence at nasopharyngeal/oropharyngeal swabs (NPS/OPS) by polymerase chain reaction after reverse transcription (RT-PCR) and for nucleoprotein-specific IgG(N) SARS-CoV-2 serology. 

Patients with prior SARS-CoV-2 infection were included 15 ± 2 days after negative response for SARS-CoV-2 detection at NPS/OPS by RT-PCR. All patients had not been previously vaccinated for SARS-CoV-2.

SARS-CoV-2 nucleic acid detection was carried out in tear samples of all participants by RT-PCR at the time of inclusion.

The study was prospectively reviewed by the Ethics Committee of the Sapienza University of Rome (code 5847 21/07/2020). The research followed the Tenets of the Declaration of Helsinki, and informed consent was obtained from all subjects of the study. All subjects were recruited and evaluated at the Department of Sense Organs, Sapienza University of Rome. 

Clinical history; complete ophthalmological and oropharyngeal examination; and serum, tear, and saliva sampling were performed. All subjects did not show the presence of ocular and/or oropharyngeal pathologies that contraindicated the collection of tears or saliva. 

Tear samples were collected in both eyes from all subjects by imbibition of sterile sharp-tip microsponges (Alcon Lab. Inc., Fort Worth, TX, USA) placed at the inferior conjunctival fornix and removed after 30 s. Tears were collected by centrifugation at 13,000× *g* for 3 min and stored at −20 °C. 

Nucleic acids were extracted and purified using the QIAamp Viral RNA Mini Kit in line with the manufacturer’s instructions. The presence of SARS-CoV-2 was investigated with a real-time RT-PCR test with a commercial kit (Applied BiosystemsTM ABI 7500 Real-Time PCR system), which targets the SARS-CoV-2 specific Orf1ab and nucleocapsid (N) gene region in clinical samples.

The results were recorded as the number of cycles threshold (Ct). The result was considered negative if Ct  >  38 (N gene) or >36 (ORF1ab gene) and positive if Ct  ≤  38 (N gene) or Ct  ≤ 36 (ORF1ab gene).

Saliva samples were collected by passive drool directly into sterile plastic tubes under the supervision of a trained provider. The samples were then centrifuged, and the resulting supernatant was transferred to other tube and stored at a temperature of −20 °C. For serum samples, peripheral venous blood was collected using a butterfly needle through a 10 mL syringe, and the serum was collected in a sterile tube. 

The presence of anti-SARS-CoV-2 IgA in the serum, tears, and saliva obtained from patients with prior SARS-CoV-2 infection and vaccinated subjects was evaluated by a CE-IVD ELISA assay (Euroimmun, Lubeck, Germany) designed to detect IgA directed against the SARS-CoV-2-S1 (including the receptor-binding domain (RBD)) of the spike protein. The used test was previously reported to have high specificity and sensitivity for the detection of IgA in serum/plasma samples, saliva, and tears [7,23]. Accordingly, serum samples were diluted 1:100, tear samples were diluted 1:5, and saliva samples were diluted 1:2 in blocking solution, and all biological fluids were processed according to the manufacturer’s instructions. IgA positivity was expressed as the ratio (R) between the OD450nm values detected in tested samples and that obtained in the calibrator sample provided by the manufacturer, representing the threshold of positivity. Each sample was assessed in duplicate. Samples were considered negative when R values were <0.8, weakly positive when R values were between 0.8 and 1.1, and strongly positive when R > 1.1.

Serum, tear, and salivary levels of IgG in all study subjects were evaluated using commercial Tecan’s SARS-CoV-2 S1/RBD IgG ELISA. It is a quantitative test detecting IgG antibodies directed against the SARS-CoV-2-S1 spike protein’s RBD. IgG positivity was expressed as U/mL.

Samples showing concentrations above the highest standard were diluted and reassayed as reported in the manufacturer’s instructions. Samples were considered negative when values were <9 U/mL, weakly positive when values were between 9 and 11, and strongly positive when values >11.

Paired sample t-test or independent sample t-test were used to compare IgG and IgA levels in serum, tear, and salivary samples at different time points and between groups (SPSS 22, Armonk, NY: IBM Corp.). Spearman’s rho test was used for the correlation between IgG and IgA levels. Data are presented as mean ± standard deviation (SD), and a *p* value < 0.05 was considered statistically significant.

## 4. Conclusions

This study demonstrated the stimulation of SARS-CoV-2-specific antibody responses at both systemic (serum) and mucosal (saliva and tears) levels after BNT162b2 vaccination and BD. Six months after primary vaccination, BNT162b2 BD was highly effective in stimulating a significant increase in systemic humoral immunity and of sIgA levels in tears, but not in saliva. However, the effectiveness of vaccination in stimulating mucosal immunity was poor when compared to mucosal immune response after SARS-CoV-2 infection. 

Our results highlight the efficacy of the available systemic immunization against COVID-19 to elicit not only the humoral immunity but also a mucosal immune reaction. However, it raises question on the effectiveness of vaccination, including the BD, to induce a valid mucosal response, supporting the potential use of topical vaccination. A larger and prospective study should be performed in order to evaluate the clinical impact of sIgA quantification in mucosal fluids and to identify potential virus-spreaders and subjects with a higher risk of relapsing infection.

## Figures and Tables

**Table 1:** Vaccinated subjects after primary BNT162b2 vaccination and at 6 months after primary BNT162b2 vaccination.

Samples	Variables	Primary BNT162b2 Vaccination	6-Months after Primary BNT162b2 Vaccination	*p*-Value
**Serum**	IgG (U/mL)	214 ± 41	55.7 ± 30.2	*p* = 0.001
	IgA (ratio sample/cal)	3.1 ± 2.3	1.5 ± 0.9	*p* = 0.001
**Saliva**	IgG (U/mL)	20.2 ± 18.6	4.3 ± 3.3	*p* = 0.001
	IgA (ratio sample/cal)	2.7 ± 1.8	1.8 ± 1	*p* = 0.032
**Tears**	IgG (U/mL)	37.1 ± 45.1	5.3 ± 7.1	*p* = 0.004
	IgA (ratio sample/cal)	2.2 ± 1.5	1.8 ± 0.8	NSS

**Table 2:** Vaccinated subjects before and after BD vaccination.

Samples	Variables	Before BD	After BD	*p*-Value
**Serum**	IgG (U/mL)	66.7 ± 31.1	219.8 ± 49	*p* < 0.001
	IgA (ratio sample/cal)	1.7 ± 1	4.5 ± 1.4	*p* < 0.001
**Saliva**	IgG (U/mL)	4.9 ± 3.8	29.3 ± 24	*p* = 0.017
	IgA (ratio sample/cal)	2.2 ± 1.2	2.7 ± 1.6	NSS
**Tears**	IgG (U/mL)	6.8 ± 9	13.7 ± 14	NSS
	IgA (ratio sample/cal)	2 ± 0.9	3.5 ± 1.5	*p* = 0.012

**Table 3:** BD vaccinated subjects vs. COVID-19 convalescents.

Samples	Variables	BD Vaccinated Subjects	COVID-19 Convalescents	*p*-Value
**Serum**	IgG (U/mL)	219.8 ± 49	95.1 ± 88.3	*p* = 0.016
	IgA (ratio sample/cal)	4.5 ± 1.4	2.8 ± 1.4	*p* = 0.030
**Saliva**	IgG (U/mL)	29.3 ± 24	4.6 ± 3.6	*p* = 0.017
	IgA (ratio sample/cal)	2.7 ± 1.6	6 ± 3.6	*p* = 0.005
**Tears**	IgG (U/mL)	13.7 ± 14	4.4 ± 3.7	NSS
	IgA (ratio sample/cal)	3.5 ± 1.5	8.5 ± 5	*p* = 0.001

## Data Availability

The data presented in this study are available on request from the corresponding author.
